# Description of three novel species of *Scandinavium*: *Scandinavium hiltneri* sp. nov., *Scandinavium manionii* sp. nov. and *Scandinavium tedordense* sp. nov., isolated from the oak rhizosphere and bleeding cankers of broadleaf hosts

**DOI:** 10.3389/fmicb.2022.1011653

**Published:** 2022-10-11

**Authors:** Daniel Maddock, Helene Kile, Sandra Denman, Dawn Arnold, Carrie Brady

**Affiliations:** ^1^Centre for Research in Bioscience, School of Applied Sciences, University of the West of England, Bristol, United Kingdom; ^2^Centre for Ecosystems, Society and Biosecurity, Forest Research, Farnham, United Kingdom; ^3^Office of the Deputy Vice-Chancellor, Harper Adams University, Newport, United Kingdom

**Keywords:** oak, rhizosphere, soil, *Scandinavium*, *Enterobacteriaceae*, *Tilia*, AOD

## Abstract

While investigating the bacterial populations of environmental samples taken from a mix of healthy and Acute Oak Decline afflicted *Quercus robur* (pedunculate or English oak) rhizosphere soil samples and swabs of bleeding lesions on *Tilia* spp. (lime) and *Quercus rubra* (red oak) trees, several strains belonging to the order *Enterobacterales* were isolated using selective media and enrichment broth. Seven strains from the *Q. robur* rhizosphere, three strains from *Tilia* spp. and one from *Q. rubra* were investigated, with their taxonomic status determined *via* a polyphasic taxonomic approach. Initially stains were identified as potential members of the recently described genus *Scandinavium*, based on the partial sequencing of three housekeeping genes. Further analysis of phenotypic traits, including fatty acid profiles, coupled with 16S rRNA gene and phylogenomic analysis of whole genome sequences were applied to a subset of the strains. Phylogenetic and phylogenomic analysis repeatedly placed the isolates in a monophyletic clade within *Scandinavium*, with four distinct clusters observed, one of which corresponded to *Scandinavium goeteborgense*, the type species of the genus. The remaining three clusters could be phenotypically and genotypically differentiated from each other and *S. goeteborgense.* As such, we describe three novel species of the genus, for which we propose the names *Scandinavium hiltneri* sp. nov. (type strain H11S7^T^ = LMG 32612^T^ = CCUG 76179^T^), *Scandinavium manionii* sp. nov. (type strain H17S15^T^ = LMG 32613^T^ = CCUG 76183^T^) and *Scandinavium tedordense* sp. nov. (type strain TWS1a^T^ = LMG 32614^T^ = CCUG 76188^T^). Additionally, the descriptions of the genus *Scandinavium* and the type species, *S. goeteborgense*, are emended.

## Introduction

Acute Oak Decline (AOD) is a disease currently threatening native and non-native oaks in Great Britain (Denman et al., 2014; Crampton et al., 2022). However, in recent years, AOD has been reported as afflicting several oak species in other countries such as Iran, Switzerland and Portugal (Moradi-Amirabad et al., 2019; Ruffner et al., 2020; Fernandes et al., 2022). AOD is a specific type of tree disease known as a Decline, which is affected by predisposing, contributing and inciting factors (Manion, 1981; Denman et al., 2022), and has undergone intensive study to understand how these interactions lead to the development of this debilitating and often fatal disease (Brady et al., 2017; Doonan et al., 2019). With the rapid increase of occurrence and distribution seen in recent years, this disease has become a looming threat to forests. By increasing our understanding of the features contributing to tree health, potential remedies to alleviate the disease may be discovered. For example asymptomatic oaks have been seen to have higher abundances of bacteria capable of ammonia-oxidation (Scarlett et al., 2021), and the addition of these bacteria to the soil has been proposed to alleviate stress in diseased oak.

Initially, the focus of the present study was to identify species in the microbial component of the oak rhizosphere as it is known to differ between healthy and diseased oaks (Pinho et al., 2020), and to identify potentially positive/detrimental bacteria that could affect oak health and therefore the development of AOD through rhizosphere interactions. Several potential enteric pathogens were identified, that were isolated from healthy and diseased *Quercus robur* rhizosphere soil from Hatchlands Park, Guildford, United Kingdom. In a separate study, aimed at identifying bacteria from bleeding cankers of broadleaf hosts in the United Kingdom, several bacteria were isolated that could not be conclusively assigned to an existing species. These strains were tentatively identified as belonging to the genus *Scandinavium* (Marathe et al., 2019) by partial 16S rRNA gene sequencing.

The order *Enterobacterales* is comprised of Gram-negative, facultative anaerobic, oxidase negative, catalase positive, rod-shaped bacteria. Although the order has existed for over 85 years, the last 20 years have proved the notably exciting, with molecular methods expanding the number of enteric bacterial species with validly published names and with that our understanding of this clinically and environmentally important order (Janda and Abbott, 2021). An important result of this has been the division of the former family *Enterobacteriaceae* into seven different families (Adeolu et al., 2016). One of the more recent additions to the order, is a monospecific genus of the updated family *Enterobacteriaceae*, with the type species *Scandinavium goeteborgense,* described in 2019 (Marathe et al., 2019). The novel genus and species were described based on a single strain isolated from a wound of a patient in the Sahlgrenska University hospital, Gothenburg, Sweden. *S. goeteborgense* is acknowledged as a species of clinical importance due to its point of isolation and it carries an interesting quinolone resistant gene variant, which could confer five-fold ciprofloxacin inhibition. This is of little surprise given the growing number of clinically significant, and multi-drug resistant, *Enterobacterales* seen in recent years (Marathe et al., 2019; Janda, 2020; Janda and Abbott, 2021).

Multigene phylogenetic and phylogenomic analyses showed *S. goeteborgense* to form a distinct lineage within the *Enterobacteriaceae* (Marathe et al., 2019). However the extent of the heterogeneity of the species is not clear due to its current single strain status. In the present study, enteric bacteria isolated from *Q*. *robur* rhizosphere soil and bleeding cankers were found to show high 16S rRNA gene sequence similarity to *S. goeteborgense*. Following a polyphasic taxonomic approach, several isolates were classified as belonging to *S. goeteborgense* and three novel *Scandinavium* species were identified from environmental sources. Here, we propose the names *Scandinavium hiltneri* sp. nov., *Scandinavium manionii* sp. nov. and *Scandinavium tedordense* sp. nov. for these three novel species, as well as the emendation of the description of the genus *Scandinavium* and the type species, *S. goeteborgense*.

## Materials and methods

### Isolation and ecology

Strains TWS1a^T^ andTWS1c were isolated from bleeding lesions of *Tilia* x *europaea* (Common Lime; Tidworth, Wiltshire, United Kingdom), while SB 3.3 was isolated from a bleeding lesion on *Tilia* x *moltkei* (Lime hybrid; Westonbirt Arboretum, Gloucestershire, United Kingdom), United Kingdom; BAC 14–01-01 was isolated from a bleeding lesion of *Q. rubra* (Knole Park, Kent, United Kingdom). Samples were taken from bleeding lesions on *Tilia* spp. and *Q. rubra* using sterile swabs, which were suspended in ¼ strength Ringers (Oxoid) solution, plated on Luria-Bertani (LB) agar, and incubated anaerobically for 48 h at 35°C (Kile et al., 2022).

The remaining strains included in this study were isolated from rhizosphere soil sampled within a 2 m radius of two *Q*. *robur* suffering from AOD and one healthy *Q*. *robur* at Hatchlands Park, Guildford, United Kingdom. Rhizosphere samples were confirmed as originating from an *Q*. *robur* root by amplification of the actin gene in a Loop Mediated Isothermal Amplification reaction (Bridget Crampton, personal communication). Soil was separated from roots, and debris removed by passing soil through a 2 cm sieve. Enteric bacteria were selectively cultured by enrichment of 10 g of wet sieved soil in *Enterobacteriaceae* Enrichment broth (EE broth, Oxoid) with incubation at 28°C for 48 h. Single colonies were isolated by diluting the EE broth suspension in ¼ strength Ringers solution to 10^−6^ and spread plating on the selective differential medium Eosin Methyl Blue agar (Merck), followed by incubation at 28°C for 48 h. Pure culture glycerol stocks were stored at −80°C and strains were routinely cultured on LB agar or in LB broth between 28–37°C. All strains investigated in this study are listed in Supplementary Table S1.

### Genotypic characterisation

DNA was extracted using the alkalic lysis method (Niemann et al., 1997), then stored at −20°C for use in all future PCR reactions. Multilocus sequence analysis (MLSA) by partial sequencing of the housekeeping genes *gyrB*, *infB* and *atpD* was performed on all strains isolated, using the primers and conditions described previously (Brady et al., 2008). All sequences were trimmed against a reference sequence following alignment in MEGA X v10.0 (Tamura et al., 2021) to the following lengths: *infB* – 615 bp, *atpD* – 642 bp and *gyrB* – 742 bp. Near-complete 16S rRNA gene (1,528 bp) sequencing was also performed using the primers, amplification cycles and annealing temperature of 55°C from (Coenye et al., 1999) for the proposed type strains of each new species (H11S7^T^, H17S15^T^ and TWS1a^T^). The 16S rRNA gene pairwise similarity for the type strains of each potential novel species was calculated using the EzBioCloud server (Yoon et al., 2017).

UGENE v38.1 (Okonechnikov et al., 2012) was used to generate consensus sequences for each of the genes used in this study, after which a concatenated dataset was built for the housekeeping genes. Sequence information for the closest phylogenetic neighbours was downloaded from NCBI Genbank *via* Blast (Benson et al., 2013). Smart model selection (Lefort et al., 2017) was then performed *via* the PhyML online server (Guindon and Gascuel, 2003) on the 16S rRNA gene and concatenated housekeeping gene datasets. Maximum likelihood phylogenetic analyses, with the appropriate models and 1,000 bootstrap replicates, was then performed in MEGA, with reliability of clusters displayed on branch nodes as a percentage.

### Box PCR

The genetic diversity of all novel strains included in this study, including *S. goeteborgense* CCUG 66741^T^, was assessed using the fingerprinting method BOX PCR using the BOX A1R primer and published protocol (Versalovic et al., 1991). The amplified products were separated in 1.5% agarose at 50 V for 3 h.

### Genomic features

To understand how the novel species, relate to each other and the type species of the genus at the genomic level, representative strains from each MLSA cluster were chosen for whole genome sequencing (H5W5 from Cluster 1, H17S15^T^ and SB 3.3 from Cluster 2, H11S7^T^ and BAC 14–01-01 from Cluster 3 and TWS1a^T^ from Cluster 4). DNA was extracted by enzymatic cell lysis with lysozyme and RNase A, purified on Solid Phase Reversible Immobilisation beads, followed by sequencing on the Illumina HiSeq platform by Microbes-NG (Birmingham, United Kingdom). Trimmomatic 3.0 was used to trim adapters with a sliding window quality cut-off of Q15 from reads (Bolger et al., 2014). SPAdes 3.11.1 was used for the *de novo* assembly, while Prokka 1.11 was used to annotate the assembled contigs (Bankevich et al., 2012; Seemann, 2014). Contamination of the whole genome sequences was ruled out by aligning the 16S rRNA gene sequences obtained *via* Sanger sequencing to the whole genome sequences in CodonCode v10.0.2 (CodonCode Corporation, USA). To complement the prokaryotic genome annotation pipeline (PGAP) determination of pseudogene numbers, accuracy of the whole genome sequences was assessed using Pseudofinder (Syberg-Olsen et al., 2022) to identify pseudogenes against the SwissProt database (Bairoch and Apweiler, 1997) accessed 31 August 2022.

The phylogenomic distance between strains was calculated though pairwise comparisons of genomes using Genome Blast Distance Phylogeny (GBDP) with the Type Strain Genome Server (Meier-Kolthoff et al., 2013). The intergenomic distance was calculated using 100 replications of the distance formula *d_5_* with the algorithm ‘trimming’ applied (Meier-Kolthoff et al., 2013). The calculated *d_5_* distance was then used to draw a genome caption tree with scaled branch lengths using FastME 2.1.6.1 (Lefort et al., 2015). Subtree Pruning and Regrafting was applied to the dataset, before being rooted at the midpoint (Farris, 1972).

To further understand the relationships between the potential novel species and *S. goeteborgense*, whole genome comparisons of the strains were made using *in silico* DNA – DNA hybridisation (*is*DDH), average nucleotide identity (ANI) and average amino identity (AAI). *is*DDH results were calculated using the Genome-to-Genome Distance calculator, which expresses *d_5_* values with a cut-off point <70% indicating a different species (Goris et al., 2007). ANIb values were calculated using JwspeciesWS (Richter et al., 2016), while AAI was calculated through the Genome-based distance matrix calculator from Kostas lab (Rodriguez and Konstantinidis, 2016).

### Cell imaging

Light microscopy, with an Olympus SC180 (Olympus Life Science, Tokyo, Japan) coupled with CellSens Version 1.11 microscopy imaging software was used for all cell size, morphology and motility assessments.

Transmission electron microscopy (FEI Tecnai 12,120 kV BioTwin Spirit TEM) was used to assess flagella arrangements for negatively stained novel species. Negative stains were made by floating grids on a mid-log phase bacterial suspension for 2 min, followed by triplicate washing, floating grids in a 3% uranyl acetate suspension for 30 s, another triplicate wash step after which excess liquid was wicked away and grids left to air dry.

### Cell physiology

Growth at 4, 10, 25, 28, 30, 37, 41°C was assessed in triplicate on tryptone soy agar to find a suitable temperature range for growth. Colony morphology was determined on Colombia Blood Agar (CBA) after 24 h incubation at 30°C as per the type species description (Marathe et al., 2019).

All *Scandinavium* strains were tested in triplicate for both pH and salt tolerance by inoculation of broths with mid-log range overnight cultures that were incubated for 24 h shaking at 180 rpm in a 37°C growth cabinet. The pH tolerance was tested from 4–10 in increasing increments of 1 in tryptone soy broth (TSB, Sigma) after altering the original pH using sodium acetate/acetic acid and carbonate/bicarbonate buffers. Saline-free nutrient broth (3 g l^−1^ beef extract, 5 g l^−1^ peptone) was used to assess the salt tolerance, by adjusting the salt concentration from 1–7% with the addition of 1% w/v NaCl.

### Antibiotic resistance

Antibiotic resistance of the novel species was tested against penicillin G 10 μg, penicillin V 10 μg, tetracycline 30 μg, ampicillin 10 μg, cefotaxime 10 μg, ciprofloxacin 10 μg. Mid-log range overnight cultures were used to make bacterial lawns on tryptone soy agar (TSA, Sigma) after which six antibiotic discs were applied equidistant to each another using a disc dispenser (Oxoid). Plates were incubated at 30°C for 24 h and the zone of clearance around the antibiotic disc assessed.

### Phenotypic tests

Phenotypic testing was performed on all strains using the API 20 E, ID 32 and 50 CHB/E galleries (with the exception of ID 32 for TWS1a^T^; bioMérieux), with GEN III GN/GP assays (Biolog) performed on H11S7^T^, BAC 14–01-01, H17S15^T^, SB 3.3, TWS1a^T^ and CCUG 66741^T^. All commercial assays were conducted according to the manufacturer’s instructions. The API 20 E and ID 32 E galleries were scored after 24 h incubation at 37°C. API 50 CHB/E galleries and GEN III plates were incubated at 30°C after which both were scored twice, at 24 and 48 h, and 16 h and 24 h, respectively. Bubble production in 3% v/v H_2_O_2_ was assessed for catalase activity and Kovács reagent (1% tetra-methyl-p-phenylenediamine dihydrochloride) for oxidase activity were also performed on all strains.

### Fatty acid methyl ester analysis

FERA Science Ltd. performed Fatty Acid Methyl Ester (FAME) analysis on a selected strains from each proposed novel species (H11S7^T^, BAC 14–01-01, H17S15^T^, SB 3.3 and TWS1a^T^) as well as *S. goeteborgense* CCUG 66741^T^ for reference. The Sherlock Microbial Identification System Version 6.4 (MIDI Inc.) protocol was followed, after strains were grown for 24 h on TSA at 30°C. Results were referenced against the RTSBA6 6.21 comparisons library.

### Virulence gene identification

To identify genes that could promote pathogenicity traits, whole genome sequences of CCUG 66741^T^, H5W5, H11S7^T^, BAC 14–01-01, H17S15^T^, SB3.3 and TWS1a^T^ were processed using PGAP during GenBank submission (Tatusova et al., 2016), and queried against several databases. Diamond v2.0.11.149 (Buchfink et al., 2021) was used to query annotated genomes against the Virulence factor database (VFDB; Liu et al., 2022), accessed 26 July 2022, *via* the Blast P command. To identify high sequence identity alignments between the genomes and the VFDB, a query cut-off of 97% and percentage identity equal or greater to than 50 were used (Doonan et al., 2019). For the identification of plant cell wall degrading enzymes and other virulence factors, the KEGG orthology search online tool was used (Aramaki et al., 2020). OrthoFinder was used to determine the conservation of virulence genes within the genomes by homology searching the genes identified from the VFDB (Emms and Kelly, 2019).

Further investigation of the Type VI Secretion System (T6SS) was performed using the SecReT6 v3 database, which was last updated 15 November 2021 (Li et al., 2015). The T6SS gene cluster protein database was downloaded, and annotated genomes queried using Diamond with the same parameters as above.

Finally, the presence of the novel quinolone resistance pentapeptide repeat protein QnrB96 (*qnrB*) gene identified in *S. goeteborgense* was assessed. The protein sequence of the gene was downloaded from GenBank *via* the assession number MK561856.1 and then compared to each novel genome *via* amino acid Blast through the Rapid Annotation using Subsystem Technology (RAST) server (Brettin et al., 2015).

## Results

### Genotypic characterisation

**Figure 1:**
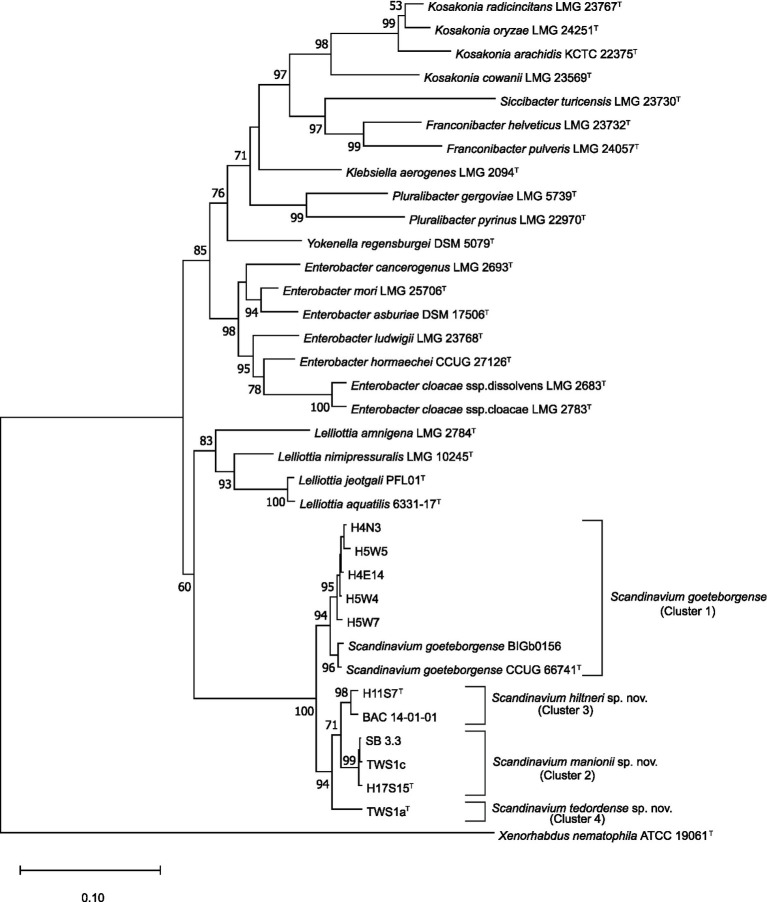
Maximum likelihood tree based on the concatenated partial gene sequences of *infB, atpD*, and *gyrB*. Sequences included are for the genus *Scandinavium*, the three proposed novel species, *Scandinavium hiltneri* sp. nov.*, Scandinavium manionii* sp. nov. and *Scandinavium tedordense* sp. nov., as well as close phylogenetic neighbours. *Xenorhabdus nematophila* (ATTCC 190601^T^) was included as the outgroup. 1000 replicate bootstrap values (> 50%) are shown at branch points. The scale bar indicates the number of substitutions per site. Type strains are denoted *via* ^T^.

The concatenated MLSA maximum likelihood phylogenetic tree (Figure 1) shows all novel strains included in the study were separated into four clusters in a single well-supported clade containing the type strain of *S. goeteborgense*. Cluster 1 constituted five strains from *Q*. *robur* rhizosphere soil, the type strain of *S. goeteborgense* (CCUG 66741^T^) and BIGb0156, isolated from rotting apple in France (Samuel et al., 2016). The high bootstrap support and minor sequence variation within this cluster suggests these strains belong to *S. goeteborgense*. Cluster 2 contained one *Q. robur* rhizosphere strain, H17S15^T^, and two strains isolated from *Tilia* spp. lesions, SB 3.3 and TWS1c. One *Q. robur* rhizosphere strain, H11S7^T^, and one strain isolated from *Q. rubra*, BAC 14–01-01, formed Cluster 3, while Cluster 4 constituted a single strain from a *Tilia* x *europaea* lesion, TWS1a^T^. The clear division from the *S. goeteborgense* cluster and high bootstrap support of Clusters 2 to 4 suggested that the strains belong to three potential novel *Scandinavium* species.

The 16S rRNA gene comparisons demonstrated high pairwise similarity percentages of all three proposed type strains to *S. goeteborgense,* with 100% completeness. TWS1a^T^ was lowest at 99.03% similar, while H17S15^T^ showed 99.80% similarity and H11S7^T^ showed 99.86% sequence similarity. Species of *Enterobacter* showed second highest pairwise similarities to the three type strains, ranging from 98.15–99.25%. Although the 16S rRNA gene comparisons indicated that the strains belong to *Scandinavium*, differentiation between any of the strains from each other and the type strain was not possible based on their 16S rRNA gene sequences. This is of little surprise as the 16S rRNA gene is poor at differentiating between species of *Enterobacteriaceae* due to its high homogeneity (Naum et al., 2008; Glaeser and Kämpfer, 2015). As such the 16S rRNA gene phylogenetic tree shown in Supplementary Figure S1 does not accurately represent the position of the novel species, with low bootstrap values and loose clustering of the proposed novel type strains with the type strain of *S. goeteborgense*.

### Box PCR

The BOX PCR results demonstrated notable levels of intra-species diversity within the samples as seen in Supplementary Figure S2. All strains belonging to one of the proposed novel species could be differentiated from each other based on their banding patterns, indicating that the novel isolates were not clonal.

### Genomic features

Genomes sizes obtained through Illumina HiSeq sequencing varied slightly by Cluster, with strains exhibiting an average size of 4.67 Mbp. The DNA G + C content reported for the genus remains consistent, ranging from 53.9 to 54.5%. The GenBank accession numbers for the genomes are JALIGB000000000 – JALIGG000000000. The Pseudofinder results identified a range of 311–419 pseudogenes present in each queried genome, which was higher than the 76–120 predicted through the NCBI PGAP. Genome features and assembly numbers are listed in Supplementary Table S2.

**Figure 2:**
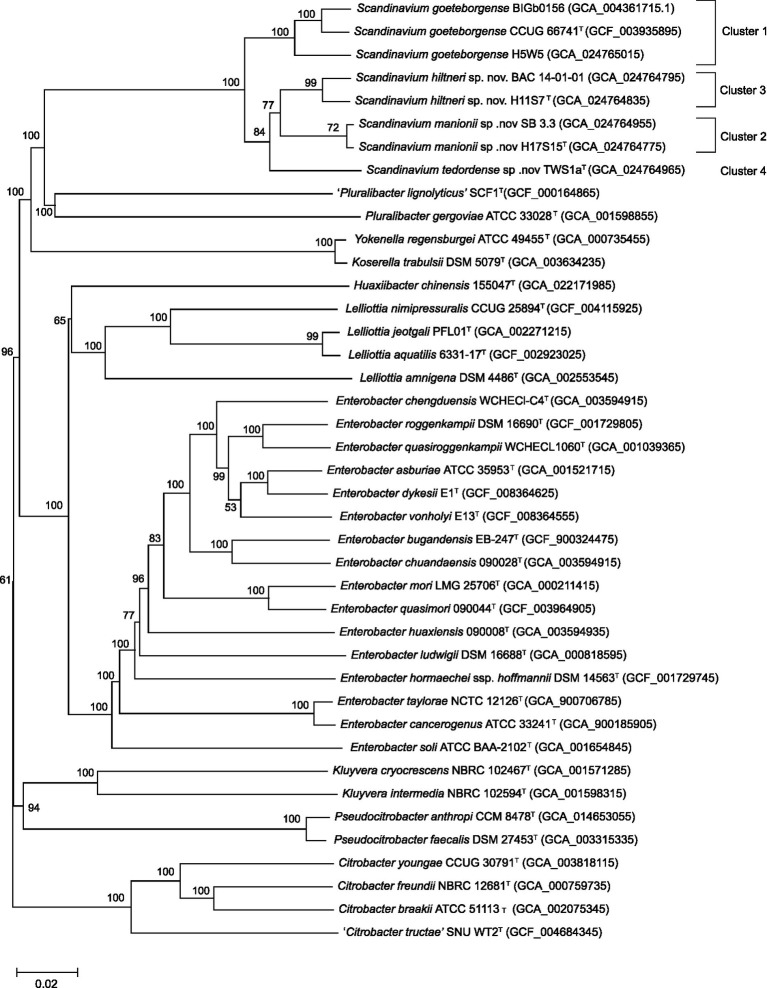
Phylogenomic tree for the genus *Scandinavium* including the three proposed novel species, *Scandinavium hiltneri* sp. nov.*, Scandinavium manionii* sp. nov. and *Scandinavium tedordense* sp. nov., and close phylogenetic neighbours. 100 replicate GBDP pseudo-bootstrap support percentages are shown (> 50%). *d*_5_ GBDP distance formula was used to scale branch lengths, and the tree was rooted at the midpoint. Type strains denoted *via* ^T^.

The resulting phylogenomic tree generated from these sequences demonstrated (Figure 2), similarly to the MLSA maximum likelihood tree, that Clusters 2–4 constitute potentially novel species within the genus *Scandinavium*. Cluster 2 (H17S15^T^ and SB 3.3), Cluster 3 (H11S7^T^ and BAC 14–01-01) and Cluster 4 (TWS1a^T^) are distinct from each other, group with no known species and have between 80 – 100% bootstrap support for each branch.

The *is*DDH and ANIb values, presented in Table 1, confirm that Clusters 2–4 constitute three novel taxa. The strains within Clusters 2 and 3 exhibited *is*DDH and ANIb values exceeding the suggested cut-off values on 70 and 95% (Konstantinidis and Tiedje, 2005), respectively, while the values between each Cluster were below these cut-off values. H5W5, a representative strain from Cluster 1, shared 69.1 and 70.2% *is*DDH similarity with the type strain of *S. goeteborgense,* CCUG 66741^T^, and with BIGb0156, respectively. These values could be considered borderline, however as the ANIb similarity between H5W5 and CCUG 66741^T^ and BIGb0156 is 95.9%, there is support for its classification as *S. goeteborgense*. The AAI values were less informative with all strains included in the comparisons showing 94–100% similarity with each other. However, despite the conserved nature of the proteins analysed, isolates assigned to the same species showed 98–100% similarity while different species could be identified by AAI values ranging from 94 to 97%, the top end of which only slightly exceeds the 96% cut-off for species delimitation (Konstantinidis and Tiedje, 2005). The resulting AAI distance clustering tree in Supplementary Figure S3 showed the same taxonomic positions as seen in the phylogenomic and MLSA trees, again supporting the description of three novel species. The names *Scandinavium hiltneri* sp. nov., *Scandinavium manionii* sp. nov. and *Scandinavium tedordense* sp. nov. are proposed for strains belonging to Clusters 2–4, respectively.

**Table 1:** Average Nucleotide Identity based on BLAST (ANIb – top right) and *in silico* DNA - DNA Hybridisation *d_5_* matrix (*is*DDH – bottom left) percentage values for novel *Scandinavium* species and *S. goeteborgense*.

ANIb *is*DDH	*S. goeteborgense* CCUG 66741^T^	*S. goeteborgense* H5W5	*S. goeteborgense* BIGb0156	*S. hiltneri* H11S7^T^	*S. hiltneri* BAC 14–01-01	*S. manionii* H17S15^T^	*S. manionii* SB 3.3	*S. tedordense* TWS1a^T^
*S. goeteborgense* CCUG 66741^T^	100	95.9	97.7	92.5	92.6	92.8	92.6	92.0
*S. goeteborgense* H5W5	69.1	100	95.9	92.6	92.7	92.9	92.7	92.0
*S. goeteborgense* BIGb0156	84.1	70.2	100	92.4	92.6	92.7	92.5	91.9
*S. hiltneri* H11S7^T^	52.0	53.3	52.1	100	97.8	94.8	94.5	92.3
*S. hiltneri* BAC 14–01-01	51.5	53.3	51.5	84.5	100	94.9	94.8	94.1
*S. manionii* H17S15^T^	52.2	53.7	52.3	63.5	62.7	100	99.4	93.7
*S. manionii* SB 3.3	52.0	53.5	52.1	62.8	62.3	96.7	100	93.4
*S. tedordense* TWS1a^T^	48.6	50.4	48.6	59	58.5	56.0	55.3	100

*Scandinavium goeteborgense* CCUG 66741^T^ (GCA_003935895), *Scandinavium goeteborgense* H5W5 (GCA_024765015), *Scandinavium goeteborgense* BIGb0156 (GCA_004361715), *Scandinavium hiltneri* H11S7^T^ (GCA_024764835), *Scandinavium hiltneri* BAC 14–01-01 (GCA_024764795), *Scandinavium manionii* H17S15^T^ (GCA_024764775), *Scandinavium manionii* SB 3.3 (GCA_024764955), *Scandinavium tedordense* TWS1a^T^ (GCA_024764965). Strains which exceed the cut off value used for species delimitation are shown in shaded boxes (>70% *is*DDH or > 95% ANI).

### Cell imaging

**Figure 3:**
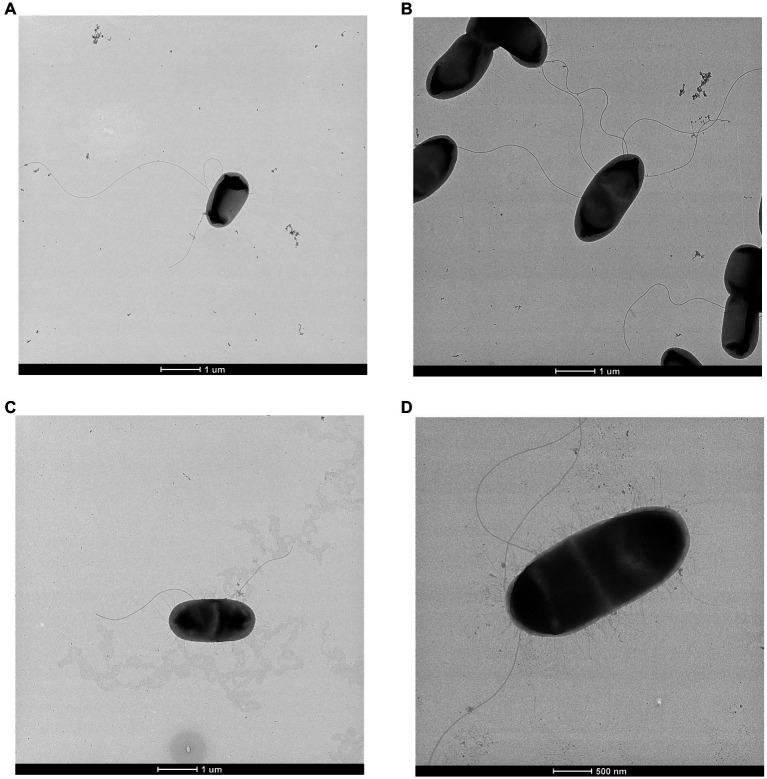
Transmission electron microscopy of **(A)** *Scandinavium goeteborgense* CCUG 66741^T^, **(B)** *Scandinavium manionii* sp. nov. H17S15^T^, **(C)** *Scandinavium hiltneri* sp. nov. H11S7^T^ and **(D)** *Scandinavium tedordense* sp. nov. TWS1a^T^ displaying their peritrichous flagella arrangement. Scale bar, 1 μm and 500 μm.

All strains were observed to be straight rods averaging 1.14 × 2.27 μm with peritrichous flagella and fimbriae. TEM images showing this are presented in Figure 3. These features are consistent with those seen for *Scandinavium* as originally described by Marathe et al. (2019).

### Cell physiology

Strains appeared as moist circular colonies with a smooth, white centre and clear rim on CBA, with colonies ranging from 1–3 mm in size. Growth was observed from 4–37°C for all strains, with no growth observed at 41°C. Growth was observed in TSB at a pH range of 6–8, while salt was tolerated in concentrations from 1–8% in NaCl-supplemented saline-free broth. All strains in this study formed agglomerated masses of growth when the salt or pH range was exceeded. All strains are oxidase negative, catalase positive and facultative anaerobic.

### Antibiotic resistance

Antibiotic resistance was recorded for penicillin V, penicillin G, ampicillin, and cefotaxime while susceptibility to ciprofloxacin and tetracycline was recorded for all strains excluding H17S15^T^, which showed low level susceptibility to ampicillin.

### Phenotypic characterisation

Differentiation of *Scandinavium* species based on phenotypic properties is quite limited with only a few discriminating features between strains isolated in this study. The most important phenotypic features for differentiation of *Scandinavium* species can be seen in Table 2. The full results for reactions to each biochemical test can be found in the protologues below and in Supplementary Table S3. The novel strains belonging to *S. goeteborgense* were phenotypically indistinguishable from CCUG 66741^T^ apart from their ability to produce acid from potassium 2-ketogluconate. *S. hiltneri* sp. nov. is phenotypically unique in its ability to utilise citrate and produce acid from sorbitol and D-raffinose. *S. manionii* sp. nov. can be phenotypically differentiated from other *Scandinavium* species by the lack of lysine decarboxylase and inability to utilise β-methyl-D-glucoside as a carbon source. *S. tedordense* sp. nov. is the only species capable of acidification of both D- and L-fucose. All novel species can be distinguished from the type strain of *S. goeteborgense* CCUG 66741^T^ by their inability to utilise γ-amino-butyric acid, α-hydroxy-butyric acid and β-hydroxy-D,L-butyric acid as carbon sources.

**Table 2:** Key phenotypic characteristics that allow for the differentiation of all known members of the genus *Scandinavium*.

Reaction	*Scandinavium goeteborgense*	*Scandinavium hiltneri*	*Scandinavium manionii*	*Scandinavium tedordense*
lysine decarboxylase	+	+	−	+
citrate utilization	−	+	−	−
sorbitol	−	+	−	−
**Fermentation of:**				
D-adonitol	−	+	+	+
dulcitol	−	V^a^	V^a^	−
D-raffinose	−	+	−	+
D-fucose	−	−	−	+
L-fucose	−	V^b^	−	+
D-arabitol	−	+	+	+
**Utilisation of:**				
β-methyl-D-glucoside	+	+	−	+
fusidic acid	+	+	+	−
myo-inositol	−	+	−	−
D-aspartic acid	+	+	−	+
D-serine	+	+	−	+
minocycline	−	+	−	−
L-pyroglutamic acid	+	+	−	−
D-saccharic acid	+	−	V^b^	+
p-hydroxy-phenylacetic acid	−	+	−	−
D-lactic acid methyl ester	+	+	−	+
tween 40	+	+	−	+
γ-amino-butyric acid	+	−	−	−
α-hydroxy-butyric acid	+	−	−	−
β-hydroxy-D,L-butyric acid	+	−	−	−
α-keto-butyric acid	−	−	−	−
acetoacetic acid	+	+	−	−

+ = positive, − = negative, V = variability within species, ^a^ = positive for type strain, ^b^ = negative for type strain. *Scandinavium goeteborgense* (*n* = 2), *Scandinavium hiltneri* (*n* = 2), *Scandinavium manionii* (*n* = 3), *Scandinavium tedordense* (*n* = 1).

### Fatty acid methyl ester analysis

The major fatty acids were identified as C_12:0_, C_14:0_, C_16:0_, C_18:1_ ω7*c,* C_17:0_ cyclo, summed feature 2 (C_14:0_ 3-OH and/or iso-C_16:1_) and summed feature 3 (C_16:1_ ω7c and/or C_16:1_ ω6c). Table 3 details the FAMEs profiles for all species of *Scandinavium*. The fatty acid profiles of all strains of all species analysed were very similar.

**Table 3:** The major fatty acid methyl ester (FAME) average % peaks and standard deviation recorded for species of *Scandinavium*.

	*Scandinavium goeteborgense*	*Scandinavium hiltneri*	*Scandinavium* *manionii*	*Scandinavium tedordense*
***Saturated fatty acids***				
C_12:0_	2.9	3.2 (± 0.18)	3.2 (± 0.14)	3.5
C_14:0_	5.2	7.5 (± 0.18)	7.0 (± 0.95)	7.4
C_16:0_	30.8	31.0 (± 0.37)	31.1 (± 0.95)	32.8
***Unsaturated fatty acids***				
C_18:1_ ω7*c*	15.6	16.5 (± 1.48)	16.2 (±0.96)	14.7
***Cyclopropane fatty acids***				
C_17:0_ cyclo	16.4	14.7 (± 0.35)	14.7 (± 4.36)	13.1
***Summed features***				
2: C_14:0_ 3-OH and/or iso-C_16:1_	7.7	7.7 (± 0.24)	7.7 (± 0.07)	8.1
3: C_16:1_ ω7*c* and/or C_16:1_ ω6*c*	17.9	16. 5 (± 0.84)	16.3 (± 3.37)	18.0

1. *Scandinavium goeteborgense* CCUG 66741^T^, 2. *Scandinavium hiltneri* (*n* = 2), 3. *Scandinavium manionii* (*n* = 2), 4. *Scandinavium tedordense* TWS1a^T^.

### Virulence genes

Using the parameters provided, concise inference to the genes present in these bacteria with results of Blast-P comparisons to the VFDB demonstrated they possess 210–237 virulence genes. Furthermore, several interesting enzymes were identified in KofamKOALA using the relevant Brite protein family identifications from the KEGG Mapper Reconstruction results. The most noteworthy enzymes identified against the databases included pectinase, adhesin/invasion protein homologues, proteins related to the assembly and utilisation of flagella and pili and the core genes required for a T6SS and associated secreted proteins. The presence of a T6SS was also identified by searching against the SecReT6, database with 156–208 different genes being aligned when querying the annotated genomes. Alignments for the membrane complex (*TssJ, TssM, TssL,* and *TagL*), baseplate (*TssK, TssF-G,* and *TssE*), spike (*PAAR* and *TssI*), sheath and tube (*TssB, TssC*, and *TssD*) and the distal end (*TssA*) were all identified with high sequence identity, namely to sequences from members of genera such as *Enterobacter*, *Klebsiella* and *Yersinia* for all strains, excluding H17S15^T^ which lacked the membrane complex. The majority of genes required for a functioning Type II Secretion System were also identified although either *gspO* or *gspS* appear to be absent. Unsurprisingly, given the type species’ clinical importance, many human diseases related proteins were also identified.

OrthoFinder assigned 34,172 genes (96.5% of total) to 4,957 orthogroups. Fifty percent of all genes were in orthogroups with 8 or more genes (G50 = 8) and were contained in the largest 2,044 orthogroups (O50 = 2,044). There were 3,222 orthogroups with all species present and 2,867 of these consisted entirely of single-copy genes. Next the virulence genes from the VFDB comparison were compared and OrthoFinder assigned 1,187 genes (99.6% of total) to 145 orthogroups. Fifty percent of all genes were in orthogroups with 8 or more genes (G50 = 8) and were contained in the largest 55 orthogroups (O50 = 55). There were 100 orthogroups with all species present and 62 of these consisted entirely of single-copy genes. This meant the virulence genes identified could be used to infer the phylogenomic position of all the species of *Scandinavium* investigated in this work, a tree representing this can be seen in Supplementary Figure S4.

Finally, Blast results from the RAST server against each novel genome demonstrated that the novel quinolone resistance pentapeptide repeat protein QnrB96 is present with high homology in all the *Scandinavium* species. The homologue in *S. hiltneri* sp. nov. showed 95% sequence identity to the complete amino acid sequence, while *S. manionii* sp. nov. showed 96% and *S. tedordense* sp. nov. showed 94% similarity. Unsurprisingly the highest homology was observed in H5W5, the strain determined to belong to *S. goeteborgense,* with 99% sequence identity. The majority of strains displayed 7–11 amino acid substitutions in the *qnrB* protein sequence when compared to the type strain of *S. goeteborgense*, excluding H5W5 which had a single substitution.

## Discussion

*Scandinavium* is a recently described monospecific genus within the family *Enterobacteriaceae* with clinical significance. When described, it was noted that single strain species descriptions are not ideal due to the limitations on phenotypic and genomic diversity (Marathe et al., 2019). The work presented here not only furthers our understanding of the genus *Scandinavium* through the addition of three new species from geographically diverse locations, but also our understanding of the type species *S. goeteborgense* by the addition of several new strains to the species. The expansion of this genus shows the range of environmental niches that *Scandinavium* occupies, with members having now been isolated from soil, plant, and human origin.

The genome-based virulence analysis indicates the presence of highly conserved virulence features which can be identified in the members of the genus *Scandinavium*. Of these, many of the genes required for a functioning T6SS and a number of effectors are included. The T6SS may provide advantages in fitness and colonisation in plants, however this diverse secretion system is not limited to causing virulence, with many commensals also possessing the assemblage (Bernal et al., 2018). Other interesting features identified in the annotated genomes include proteins closely-related to the novel quinolone resistance pentapeptide repeat protein QnrB96, previously identified from *S. goeteborgense.* The gene, which conferred a five-fold increase in the minimum inhibitory concentration of ciprofloxacin when expressed in *E. coli*, appears to be a feature of *Scandinavium* species. Although the new species do display variations in the amino acid sequence potentially producing novel variants, all strains were susceptible to 10 μg ciprofloxacin *in vitro*. The genomes also contained a relatively high number of pseudogenes which, along with the identified virulence factors, may implicate the isolates as pathogens. Pseudogenes are most commonly associated with endosymbionts or pathogens that have rapidly changed their ecology, rendering high proportions of their genes unnecessary (Ochman and Davalos, 2006; Goodhead and Darby, 2015). Virulence factor identification alone is not enough to implicate an organism as a pathogen. However, the fact that most of the novel strains in this study have been isolated from either bleeding lesions on broadleaf hosts, or from the roots of *Q*. *robur* suffering from AOD, poses an interesting implication regarding plant health. Further investigation of this recently described and expanding genus is warranted to understand the potential of its members as plant pathogens.

Based on the genomic, genotypic, chemotaxonomic and phenotypic data obtained, we conclude that the strains examined in this study represent three novel species, for which we propose the names *Scandinavium hiltneri* sp. nov. (type strain = H11S7^T^), *Scandinavium manionii* sp. nov. (type strain = H17S15^T^) and *Scandinavium tedordense* sp. nov. (type strain = TWS1a^T^). Emendments of the genus and type species descriptions are also presented.

### Emendation of the genus *Scandinavium*

*Scandinavium* (Scan.di.na’vi. um. N.L. neut. n. *Scandinavium* genus named after Scandinavia: the European peninsula where the type strain of the type species was isolated and characterised).

This description is based on the data from (Marathe et al., 2019) and this study.

Gram-negative straight rods which are 1–1.3 × 1.9–2.7 μm, motile by peritrichous flagella and possess fimbriate. Facultative anaerobic, oxidase negative and catalase positive. The colonies appear as moist, white circles with clear rims on CBA averaging 1–3 mm in size. Growth is observed at 4–37°C with an optimum growth temperature of 30°C; the salt and pH range for growth are 1–7% and 6–8, respectively. Outside of the salt range, coagulated masses of growth can be observed in broth. Positive for β-galactosidase. Negative for arginine dihydrolase, ornithine decarboxylase, H_2_S production, urease, tryptophan deaminase, indole production, acetoin production and gelatinase. Nitrate is reduced to nitrite. Acid is produced from: glucose, mannitol, amygdalin, L-arabinose, glycerol, D-ribose, D-xylose, D-galactose, D-fructose, D-mannose, methyl-αD-glucopyranoside, N-acetylglucosamine, arbutin, esculin ferric citrate, salicin, D-cellobiose, D-maltose, D-lactose, D-trehalose, gentiobiose and potassium gluconate (API 20and 50 CHB/E). Acid is produced from galacturonate and β-glucosidase is present (ID32). Utilises the following carbon sources: dextrin, D-salicin, N-acetyl-D-glucosamine, N-acetyl-β-D-mannosamine, N-acetyl neuraminic acid, 3-methyl glucose, inosine, D-glucose-6-phosphate, D-fructose-6-phosphate, glycyl-L-proline, L-alanine, L-arginine, L-aspartic acid, L-glutamic acid, L-histidine, L-serine, D-galaturonic acid, L-galactonic acid lactone, D-gluconic acid, D-glucuronic acid, glucuronamide, quinic acid, methyl pyruvate, L-lactic acid, citric acid, L-malic acid, bromo-succinic acid and acetic acid. Resistant to 1% sodium lactate, D-serine, troleandomycin, rifamycin, lincomycin, guanidine hydrochloric acid, niaproof 4, vancomycin, tetrazolium violet, tetrazolium blue, nalidixic acid, lithium chloride, aztreonam and sodium butyrate (Biolog Gen III). Variable for: lysine decarboxylase, citrate utilization and fermentation of sorbitol, rhamnose, melibiose, D-adonitol, dulcitol, D-raffinose, D-turanose, D-fucose, L-fucose, D-arabitol and potassium 2-ketogluconate; acidification of phenol red, palatinose and production of malonate, α-glucosidase, α-galactosidase. Utilisation of the following carbon sources is variable: sucrose, stachyose, β-methyl-D-glucoside, N-acetyl-D-galactosamine, myo-inositol, D-aspartic acid, L-pyroglutamic acid, mucic acid, D-saccharic acid, p-hydroxy-phenylacetic acid, D-lactic acid methyl ester, tween 40, γ-amino-butyric acid, α-hydroxy-butyric acid, β-hydroxy-D,L-butyric acid and acetoacetic acid; susceptibility to fusidic acid, D-serine and minocycline also varies. The major fatty acids are C_12:0_, C_14:0_, C_16:0_, C_18:1_ ω7*c,* C_17:0_ cyclo, summed features 2 (C_14:0_ 3-OH and/or iso-C_16:1_) and summed features 3 (C_16:1_ ω7*c* and/or C_16:1_ ω6*c*). The DNA G + C content ranges from 53.9–54.5%.

Strains have been isolated from a range of sources, including human wound infection, rhizosphere soil and bleeding lesions of broadleaf hosts.

The type species is *Scandinavium goeteborgense* (CCUG 66741^T^ = CECT 9823^T^ = NCTC 14286^T^).

### Description of *Scandinavium hiltneri* sp. nov.

*Scandinavium hiltneri* (hilt’ne.ri. N.L. gen. n. *hiltneri*, named in honour of Lorenz Hiltner, the scientist who coined the term ‘rhizosphere’ in 1904, from where the majority of original isolates were obtained).

The species shares the major characteristics of the genus. Gram-negative motile rods (1.08–1.19 × 1.96–2.41 μm) that occur singly. Colonies appear as moist, raised, white circles with clear smooth margins on CBA, averaging 1–2 mm in size. Positive for lysine decarboxylase and citrate utilisation. Acid is produced from: sorbitol, rhamnose, D-adonitol, D-raffinose, D-arabitol, potassium 2-ketogluconate (API 20/ 50 CHB/E). Positive for production of α-galactosidase and acidification of phenol red (ID32). The following carbon sources are utilised: sucrose, stachyose, β-methyl-D-glucoside, N-acetyl-D-galactosamine, myo-inositol, D-aspartic acid, D-serine, L-pyroglutamic acid, p-hydroxy-phenylacetic acid, D-lactic acid methyl ester, tween 40 and acetoacetic acid. Resistant to fusidic acid and minocycline (Biolog Gen III). Variable for the fermentation of melibiose, dulcitol, D-turanose, L-fucose and utilisation of the carbon source mucic acid.

The DNA G + C content of the type strain is 54.2 mol %.

The type strain H11S7^T^ (= LMG 32612^T^ = CCUG 76179^T^) was isolated from *Quercus robur* rhizosphere soil in Hatchlands, Guildford, United Kingdom.

### Description of *Scandinavium manionii* sp. nov.

*Scandinavium manionii* (ma.ni.o’ni. i. N.L. gen. n. *manionii*, named after Paul Manion, who defined the decline disease spiral furthering our understanding of the range of influences on forest diseases).

The species shares the major characteristics of the genus. Gram negative motile rods (1.12–1.18 × 2.11–2.45 μm) which occur singly. Colonies appear as moist, raised, white circles with clear smooth margins on CBA, averaging 2–3 mm in size. On TSA strains have a dried-out brittle appearance which allows visual differentiation from other species of *Scandinavium.* Negative for lysine decarboxylase and citrate utilisation. Acid is produced from D-adonitol, D-arabitol and potassium 2-ketogluconate (API 20 and 50 CHB/E). Resistant to fusidic acid (Biolog Gen III). Variable for fermentation of rhamnose, dulcitol, acidification of phenol red, palatinose and production of malonate and α-glucosidase. Utilisation of the following carbon sources is variable: N-acetyl-D-galactosamine, mucic acid, D-saccharic acid and p-hydroxy-phenylacetic acid.

The DNA G + C content of the type strain is 53.9 mol %.

The type strain H17S15^T^ (= LMG 32613^T^ = CCUG 76183^T^) was isolated from *Quercus robur* rhizosphere soil in Hatchlands, Guildford, United Kingdom.

### Description of *Scandinavium tedordense* sp. nov.

*Scandinavium tedordense* (te.dor.den’se. M.L. neut. n. *tedordense*, pertaining to Tedorde, the medieval name of Tidworth, where the type strain was isolated).

The species shares the major characteristics of the genus. Gram negative motile rods (1 × 2.3–2.5 μm) which occur singly. Colonies appear as moist, raised, white circles with clear smooth margins on CBA, averaging 1–2 mm in size. Positive for lysine decarboxylase. Acid is produced from rhamnose, D-adonitol, D-raffinose, D-turanose, D-fucose, L-fucose, D-arabitol and potassium 2-ketogluconate (API 20 and 50 CHB/E). Utilises the following carbon sources: β-methyl-D-glucoside, N-acetyl-D-galactosamine, D-aspartic acid, D-serine, mucic acid, D-saccharic acid, D-lactic acid methyl ester and tween 40 (Biolog Gen III).

The DNA G + C content of the type strain is 53.9%.

The type strain TWS1a^T^ (= LMG 32614^T^ = CCUG 76188^T^) was isolated from a bleeding lesion on a *Tilia* x *europaea* tree in Tidworth, Wiltshire, United Kingdom.

### Emendation of the description of the species *Scandinavium goeteborgense*

The description follows that of (Marathe et al., 2019) with the following additions.

Positive for lysine decarboxylase. Acid is produced from sorbitol and D-turanose (API 20/50 CHB/E). Acidifies phenol red (ID32). Utilises the following carbon sources: β-methyl-D-glucoside, N-acetyl-D-galactosamine, D-aspartic acid, D-serine, L-pyroglutamic acid, D-saccharic acid, tween 40, γ-amino-butyric acid, α-hydroxy-butyric acid, β-hydroxy-D,L-butyric acid and acetoacetic acid. Resistant to fusidic acid. Variable for the fermentation of potassium 2-ketogluconate (Biolog Gen III).

The DNA G + C content of the type strain is 54.3%.

## Data availability statement

The data presented in the study are deposited in GenBank/EMBL/DDBJ under the accession numbers: OM987267 – OM987269 (16S rRNA); ON221904 – ON221914 (infB); ON221882 – ON221892 (atpD); ON221893 – ON221902 (gyrB); JALIGB000000000 – JALIGG000000000 (whole genome). The raw whole genome sequencing data generated in this study has been deposited in the NCBI Sequence Read Archive (SRA) under the accession numbers SRR21315071 - SRR21315076.

## Author contributions

DM was involved in the conceptualization, data curation, formal analysis, investigation, methodology, validation, visualization, writing, reviewing and editing of the work. HK contributed to the investigation and methodology. CB was involved in the provision of resources and the conceptualization, writing, reviewing, and editing of the manuscript. DA and SD were responsible for funding acquisition and the reviewing and editing of the manuscript. All authors contributed to the article and approved the submitted version.

## Funding

This research was supported by Woodland Heritage (Charity no. 1041611) and the University of the West of England. CB, SD, and DA received support from the UK Research and Innovation’s (UKRI) Strategic Priorities Fund (SPF) programme on Bacterial Plant Diseases (grant BB/T010886/1) funded by the Biotechnology and Biological Sciences Research Council (BBSRC), the Department for Environment, Food and Rural Affairs (Defra), the Natural Environment Research Council (NERC) and the Scottish Government. Genome sequencing was provided by MicrobesNG (http://www.microbesng.uk) which is supported by the BBSRC (grant number BB/L024209/1). The authors also thank Action Oak for their support.

## Conflict of interest

The authors declare that the research was conducted in the absence of any commercial or financial relationships that could be construed as a potential conflict of interest.

## Publisher’s note

All claims expressed in this article are solely those of the authors and do not necessarily represent those of their affiliated organizations, or those of the publisher, the editors and the reviewers. Any product that may be evaluated in this article, or claim that may be made by its manufacturer, is not guaranteed or endorsed by the publisher.
