# Supplementary material for: Description of three novel species of Scandinavium: Scandinavium hiltneri sp. nov., Scandinavium manionii sp. nov. and Scandinavium tedordense sp. nov., isolated from the oak rhizosphere and bleeding cankers of broadleaf hosts
Source: Front Microbiol. 2022 Oct 11;13:1011653. doi: 10.3389/fmicb.2022.1011653 (PMC9592992; doi:10.3389/fmicb.2022.1011653)
Supplement: Supplementary file 1 [file Data_Sheet_1.docx]

Supplementary Material

**Suppl. Fig. S1:** 16S rRNA gene maximum likelihood phylogenetic tree for *Scandinavium* species, the proposed novel species and their closest phylogenetic neighbours. The near complete (1,346 bp) 16S rRNA gene sequences were used, 1000 bootstrap replicate percentage values (> 50 %) are shown at the nodes and the scale bar indicates the number of nucleotide substitutions per site. The outgroup is *Plesiomonas shigelloides* NCIMB 9242^T^. GenBank accession numbers shown in parentheses and ^T^ = type strain.


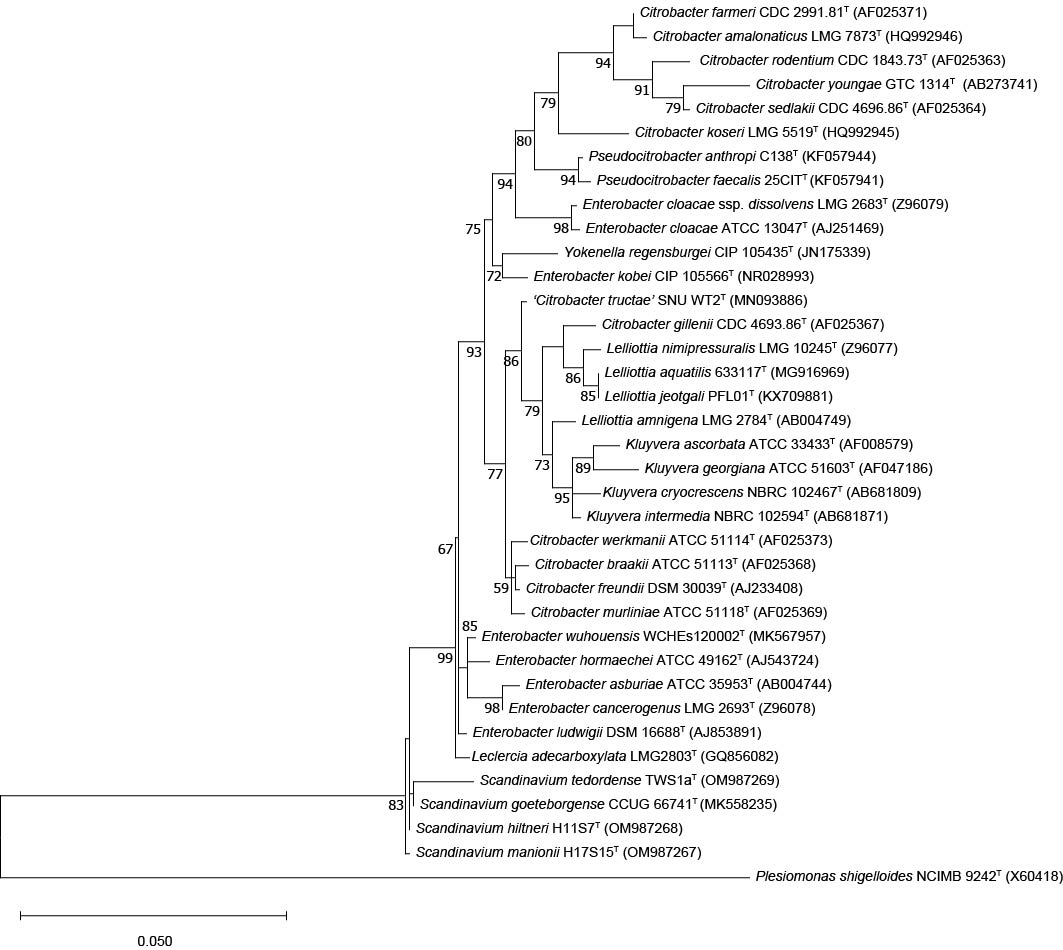


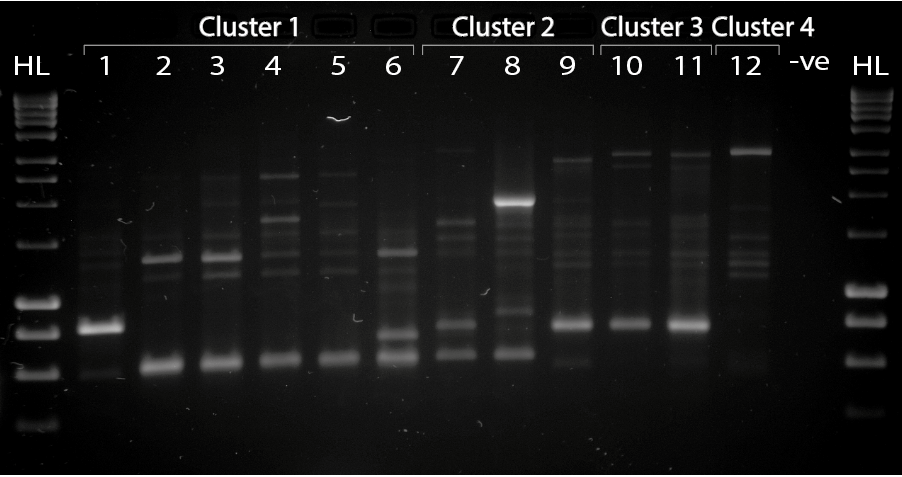
**Suppl. Fig. S2:** BOX PCR fingerprinting patterns generated from strains of *Scandinavium* *goeteborgense* and the three proposed novel species of *Scandinavium.*

(1) *Scandinavium goeteborgense* CCUG 66741^T^, (2) *Scandinavium goeteborgense* H5W7, (3) *Scandinavium goeteborgense* H4E14, (4) *Scandinavium goeteborgense* H5W4, (5) *Scandinavium goeteborgense* H4N3, (6) *Scandinavium goeteborgense* H5W5, (7) *Scandinavium manionii* H17S15^T^, (8) *Scandinavium manionii* TWS1c (19) *Scandinavium manionii* SB 3.3, (10) *Scandinavium hitlneri* H11S7^T^, (11) *Scandinavium hiltneri* BAC 14-01-01 (12) *Scandinavium tedordense* TWS1a^T^. -ve is negative control. A 1Kb Hyperladder (Bioline) was run as a size marker in both the first and last wells.

**Suppl. Fig S3:** Average Amino Acid Identity tree for species of *Scandinavium*. The tree indicates the presence of four unique clusters which represent the type species *Scandinavium goeteborgense* and three proposed novel species. Scale bar indicates the number of substitutions per site.


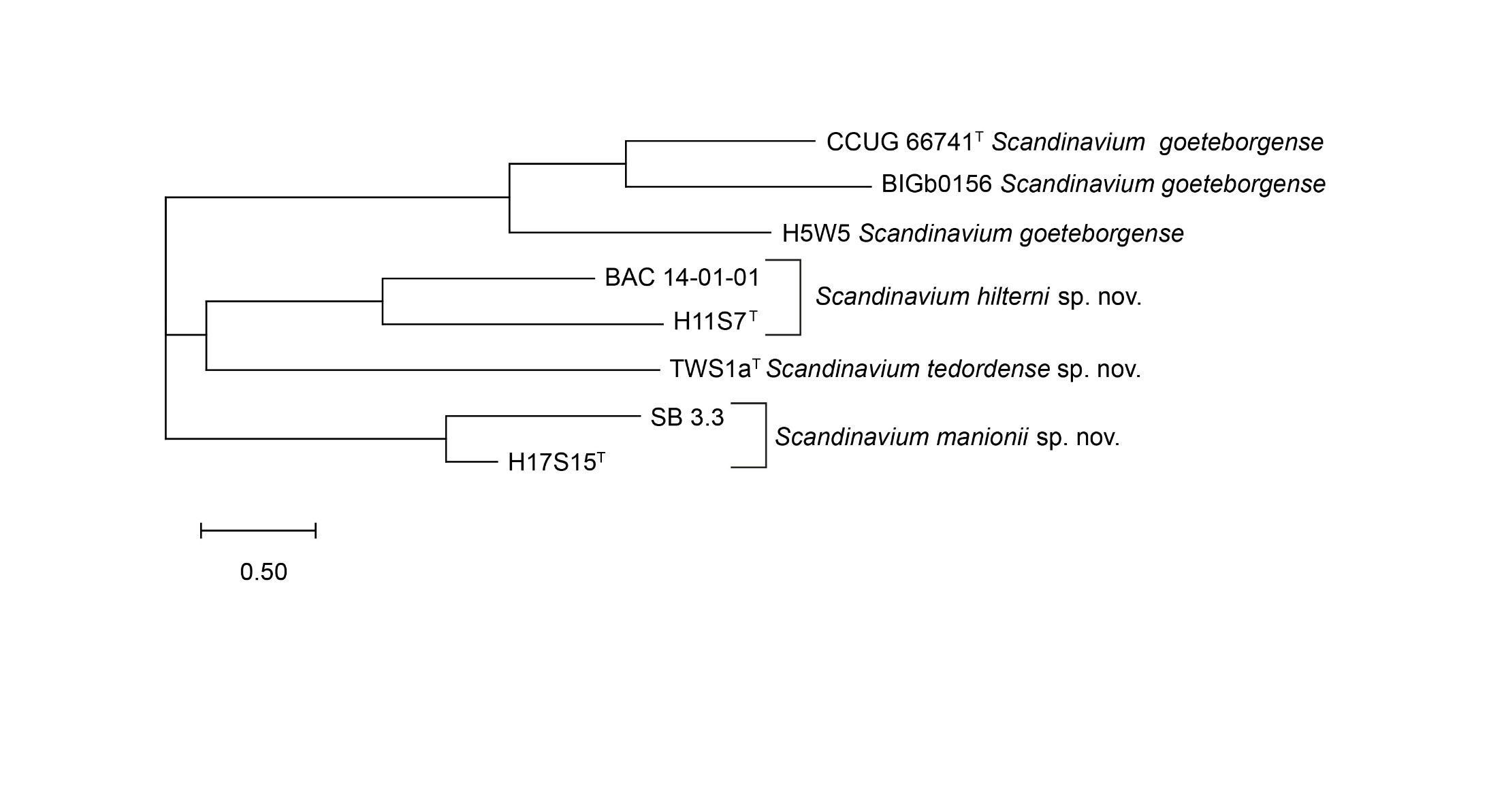


**Suppl. Fig. S4:** **(A)** Unrooted whole genome phylogenetic tree based of the conserved orthologous gene groupings found in each representative cluster of *Scandinavium*. (**B)** Unrooted phylogenetic tree based of the conserved orthologous predicted virulence gene groupings found in each representative cluster of *Scandinavium*. Both trees indicate the presence of four unique clusters which represent the type species *Scandinavium goeteborgense* and three proposed novel species. However, B shows poor support for Cluster 2 due to the reduced number of virulence genes identified in H17S15^T^

**(A)**


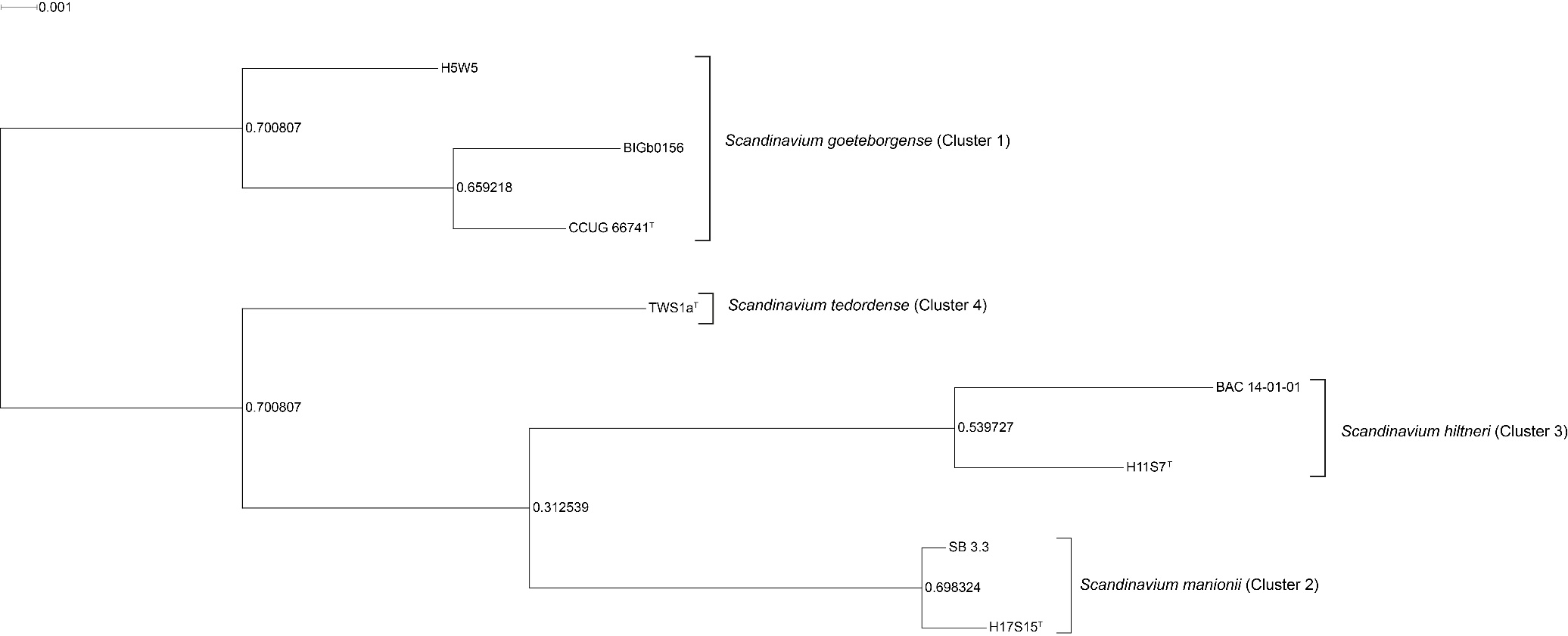


**
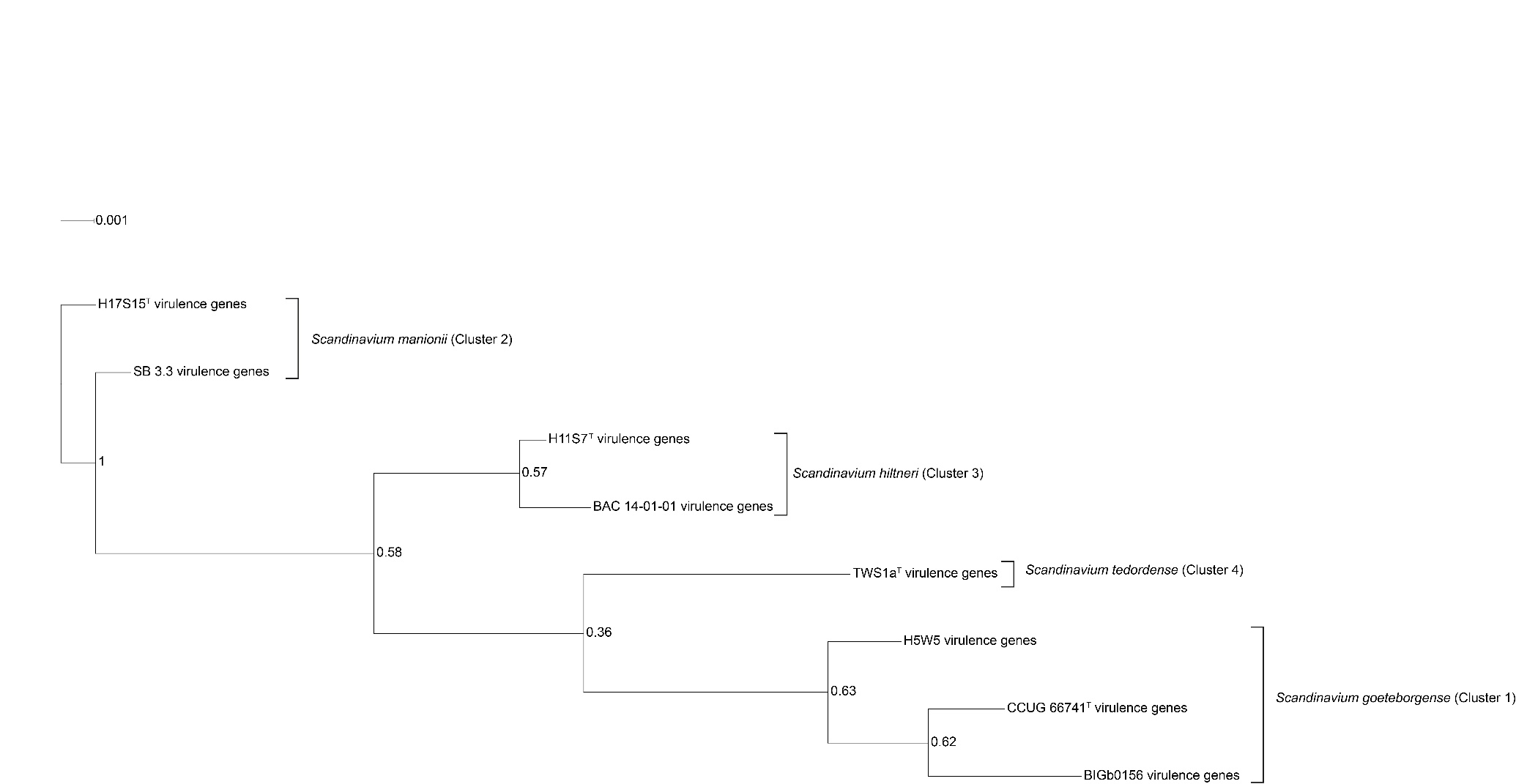
**

**(B)**

Suppl. Table S1: Strains investigated in this study.

| **GenBank accession numbers** | | |  |  |  |  |
| --- | --- | --- | --- | --- | --- | --- |
| **Strain** | **Location** | **Year of isolation** | **Source** | ***infB**** | ***atpD**** | ***gyrB**** |
| ***Scandinavium goeteborgense*** |  |  |  |  |  |  |
| H5W7 | Hatchlands, Guildford, UK | 2020 | *Quercus robur* (AOD) rhizosphere soil | ON221910 | ON221888 | ON221899 |
| H5W4 | Hatchlands, Guildford, UK | 2020 | *Quercus robur* (AOD) rhizosphere soil | ON221912 | ON221890 | ON221901 |
| H4E14 | Hatchlands, Guildford, UK | 2020 | *Quercus robur* (healthy) rhizosphere soil | ON221911 | ON221889 | ON221900 |
| H4N3 | Hatchlands, Guildford, UK | 2020 | *Quercus robur* (healthy) rhizosphere soil | ON221913 | ON221891 | ON221902 |
| H5W5 | Hatchlands, Guildford, UK | 2020 | *Quercus robur* (AOD) rhizosphere soil | ON221914 | ON221892 | ON221903 |
| ***Scandinavium hiltneri*** |  |  |  |  |  |  |
| H11S7^T^ | Hatchlands, Guildford, UK | 2020 | *Quercus robur* (AOD) rhizosphere soil | ON221904 | ON221882 | ON221893 |
| BAC 14-01-01 | Knole Park, Kent, UK | 2020 | Bleeding lesion on *Quercus rubra* | ON221905 | ON221883 | ON221894 |
| ***Scandinavium manionii*** |  |  |  |  |  |  |
| H17S15^T^ | Hatchlands, Guildford, UK | 2020 | *Quercus robur* (AOD) rhizosphere soil | ON221906 | ON221884 | ON221895 |
| SB 3.3 | Westonbirt arboretum, Gloucestershire | 2021 | Bleeding lesion on *Tilia x moltkei* | ON221908 | ON221886 | ON221897 |
| TWS1c | Tidworth, Wiltshire, UK | 2021 | Bleeding lesion on *Tilia x europaea* | ON221907 | ON221885 | ON221896 |
| ***Scandinavium tedordense*** |  |  |  |  |  |  |
| TWS1a^T^ | Tidworth, Wiltshire, UK | 2021 | Bleeding lesion on *Tilia x europaea* | ON221909 | ON221887 | ON221898 |

Suppl. Table S2: The whole genome sequence information for strains investigated in this study.

| **Strain** | **GenBank accession** | **Biosample number** | **Size (Mbp)** | **Number of contigs** | **N50** | **Mean coverage** | **Number of coding sequences** | **Number of pseudo genes**  **(PGAP /**  **Pseudofinder)** | **Numbers of RNAs** | **GC content (mol %)** |
| --- | --- | --- | --- | --- | --- | --- | --- | --- | --- | --- |
| ***Scandinavium goeteborgense*** |  |  |  |  |  |  |  |  |  |  |
| H5W5 | JALIGB000000000 | SAMN27163994 | 4.77 | 21 | 652013 | 90.7 | 4,439 | 77 / 317 | 103 | 54.5 |
| ***Scandinavium hiltneri*** |  |  |  |  |  |  |  |  |  |  |
| H11S7^T^ | JALIGE000000000 | SAMN27163997 | 4.84 | 79 | 309159 | 169.2 | 4,565 | 120 / 419 | 90 | 53.9 |
| BAC 14-01-01 | JALIGF000000000 | SAMN27163998 | 4.61 | 63 | 295467 | 143.8 | 4,299 | 110 / 364 | 96 | 54.2 |
| ***Scandinavium manionii*** |  |  |  |  |  |  |  |  |  |  |
| H17S15^T^ | JALIGC000000000 | SAMN27163995 | 4.64 | 40 | 394704 | 74.1 | 4,314 | 76 / 311 | 90 | 54.2 |
| SB 3.3 | JALIGD000000000 | SAMN27163996 | 4.39 | 47 | 439894 | 82.6 | 4,834 | 89 / 363 | 91 | 53.9 |
| ***Scandinavium tedordense*** |  |  |  |  |  |  |  |  |  |  |
| TWS1a^T^ | JALIGG000000000 | SAMN27163999 | 4.75 | 61 | 433482 | 152.6 | 4,457 | 87 / 352 | 94 | 53.9 |

Suppl. Table S3: Complete phenotypic profiles for strain investigated in this study.

|  | ***Scandinavium goeteborgense* CCUG 66741^T^** | ***Scandinavium goeteborgense* H5W5** | ***Scandinavium* *hiltneri*** **H11S7^T^** | ***Scandinavium* *hiltneri*** **BAC 14-01-01** | ***Scandinavium manionii***  **H17S15^T^** | ***Scandinavium manionii*** **SB 3.3** | ***Scandinavium manionii*** **TWS1c** | ***Scandinavium tedordense***  **TWS1a^T^** |
| --- | --- | --- | --- | --- | --- | --- | --- | --- |
| **API 20** |  |  |  |  |  |  |  |  |
| β-galactosidase (ONPG) | + | + | + | + | + | + | + | + |
| arginine dihydrolase | - | - | - | - | - | - | - | - |
| lysine decarboxylase | + | + | - | - | + | + | + | + |
| ornithine decarboxylase | - | - | - | - | - | - | - | - |
| citrate utilization | - | - | - | - | + | + | + | - |
| H2S production | - | - | - | - | - | - | - | - |
| urease | - | - | - | - | - | - | - | - |
| tryptophan deaminase | - | - | - | - | - | - | - | - |
| indole production | - | - | - | - | - | - | - | - |
| acetoin production (VP) | - | - | - | - | - | - | - | - |
| gelatinase | - | - | - | - | - | - | - | - |
| glucose | + | + | + | + | + | + | + | + |
| mannitol | + | + | + | + | + | + | + | + |
| inositol | - | - | - | - | - | - | - | - |
| sorbitol | - | - | - | - | + | + | + | - |
| rhamnose | + | + | + | - | + | + | + | + |
| saccharose | - | - | - | - | - | - | - | - |
| melibiose | - | - | - | - | - | + | + | - |
| amygdalin | + | + | + | + | + | + | + | + |
| L-arabinose | + | + | + | + | + | + | + | + |
| nitrite (NO2) | + | + | + | + | + | + | + | + |
| oxidase | - | - | - | - | - | - | - | - |
| catalase | + | + | + | + | + | + | + | + |
| **API 50 CHB/E** |  |  |  |  |  |  |  |  |
| glycerol | + | + | + | + | + | + | + | + |
| erythritol | - | - | - | - | - | - | - | - |
| D-arabinose | - | - | - | - | - | - | - | - |
| D-ribose | + | + | + | + | + | + | + | + |
| D-xylose | + | + | + | + | + | + | + | + |
| L-xylose | - | - | - | - | - | - | - | - |
| D-adonitol | - | - | + | + | + | + | + | + |
| methyl-βD-xylopyranoside | - | - | - | - | - | - | - | - |
| D-galactose | + | + | + | + | + | + | + | + |
| D-fructose | + | + | + | + | + | + | + | + |
| D-mannose | + | + | + | + | + | + | + | + |
| L-sorbose | - | - | - | - | - | - | - | - |
| dulcitol | - | - | + | - | + | - | + | - |
| methyl- αD-mannopyranoside | - | - | - | - | - | - | - | - |
| methyl-αD-glucopyranoside | + | + | + | + | + | + | + | + |
| N-acetylglucosamine | + | + | + | + | + | + | + | + |
| arbutin | + | + | + | + | + | + | + | + |
| esculin ferric citrate | + | + | + | + | + | + | + | + |
| salicin | + | + | + | + | + | + | + | + |
| D-cellobiose | + | + | + | + | + | + | + | + |
| D-maltose | + | + | + | + | + | + | + | + |
| D-lactose | + | + | + | + | + | + | + | + |
| D-trehalose | + | + | + | + | + | + | + | + |
| inulin | - | - | - | - | - | - | - | - |
| D-melezitose | - | - | - | - | - | - | - | - |
| D-raffinose | - | - | - | - | + | + | + | + |
| amidon (starch) | - | - | - | - | - | - | - | - |
| glycogen | - | - | - | - | - | - | - | - |
| xylitol | - | - | - | - | - | - | - | - |
| gentiobiose | + | + | + | + | + | + | + | + |
| D-turanose | + | + | - | - | - | - | + | + |
| D-lyxose | - | - | - | - | - | - | - | - |
| D-tagatose | - | - | - | - | - | - | - | - |
| D-fucose | - | - | - | - | - | - | - | + |
| L-fucose | - | - | - | - | - | + | - | + |
| D-arabitol | - | - | + | + | + | + | + | + |
| L-arabitol | - | - | - | - | - | - | - | - |
| potassium gluconate | + | + | + | + | + | + | + | + |
| potassium 2-ketogluconate | - | + | + | + | + | + | + | + |
| potassium 5-ketogluconate | - | - | - | - | - | - | - | - |
|  | | | | | | | | |
| **ID32** |  |  |  |  |  |  |  |  |
| galacturonate | + | + | + | + | + | + | ND | ND |
| lipase | - | - | - | - | - | - | ND | ND |
| phenol red | + | + | + | - | + | + | ND | ND |
| β-glucosidase | + | + | + | + | + | + | ND | ND |
| palatinose | - | - | - | + | - | - | ND | ND |
| β-glucuronidase | - | - | - | - | - | - | ND | ND |
| malonate | - | - | - | + | - | - | ND | ND |
| N-acetyl-β-glucosaminidase | - | - | - | - | - | - | ND | ND |
| α-glucosidase | - | - | - | + | - | - | ND | ND |
| α-galactosidase | - | - | - | - | + | + | ND | ND |
| α-maltosidase | - | - | - | - | - | - | ND | ND |
| L-aspartic acid arylamidase | - | - | - | - | - | - | ND | ND |
| **Biolog GEN III GN/GP** |  |  |  |  |  |  |  |  |
| dextrin | + | ND | + | + | + | + | ND | + |
| sucrose | + | ND | - | - | + | + | ND | - |
| stachyose | + | ND | - | - | + | + | ND | - |
| positive control | + | ND | + | + | + | + | ND | + |
| pH 6 | + | ND | + | + | + | + | ND | + |
| pH 5 | + | ND | + | + | + | + | ND | + |
| β-methyl-D-glucoside | + | ND | - | - | + | + | ND | + |
| D-salicin | + | ND | + | + | + | + | ND | + |
| N-acetyl-D-glucosamine | + | ND | + | + | + | + | ND | + |
| N-acetyl-β-D-mannosamine | + | ND | + | + | + | + | ND | + |
| N-acetyl-D-galactosamine | + | ND | - | + | + | + | ND | + |
| N-acetyl neuraminic acid | + | ND | + | + | + | + | ND | + |
| 1% NaCl | + | ND | + | + | + | + | ND | + |
| 4% NaCl | + | ND | + | + | + | + | ND | + |
| 8% NaCl | - | ND | + | + | + | + | ND | + |
| 3-methyl glucose | + | ND | + | + | + | + | ND | + |
| inosine | + | ND | + | + | + | + | ND | + |
| 1% sodium lactate | + | ND | + | + | + | + | ND | + |
| fusidic acid | + | ND | + | + | + | + | ND | - |
| D-serine | + | ND | + | + | + | + | ND | + |
| myo-inositol | - | ND | - | - | + | + | ND | - |
| D-glucose-6-phosphate | + | ND | + | + | + | + | ND | + |
| D-fructose-6-phosphate | + | ND | + | + | + | + | ND | + |
| D-aspartic acid | + | ND | - | - | + | + | ND | + |
| D-serine | + | ND | - | - | + | + | ND | + |
| Troleandomycin | + | ND | + | + | + | + | ND | + |
| Rifamycin | + | ND | + | + | + | + | ND | + |
| Minocycline | - | ND | - | - | + | + | ND | - |
| gelatin | - | ND | - | - | - | - | ND | - |
| glycyl-L-proline | + | ND | + | + | + | + | ND | + |
| L-alanine | + | ND | + | + | + | + | ND | + |
| L-arginine | + | ND | + | + | + | + | ND | + |
| L-aspartic acid | + | ND | + | + | + | + | ND | + |
| L-glutamic acid | + | ND | + | + | + | + | ND | + |
| L-histidine | + | ND | + | + | + | + | ND | + |
| L-pyroglutamic acid | + | ND | - | - | + | + | ND | - |
| L-serine | + | ND | + | + | + | + | ND | + |
| Lincomycin | + | ND | + | + | + | + | ND | + |
| Guanidine HCl | + | ND | + | + | + | + | ND | + |
| Niaproof 4 | + | ND | + | + | + | + | ND | + |
| pectin | - | ND | - | - | - | - | ND | - |
| D-galaturonic acid | + | ND | + | + | + | + | ND | + |
| L-galactonic acid lactone | + | ND | + | + | + | + | ND | + |
| D-gluconic acid | + | ND | + | + | + | + | ND | + |
| D-glucuronic acid | + | ND | + | + | + | + | ND | + |
| glucuronamide | + | ND | + | + | + | + | ND | + |
| mucic acid | + | ND | - | + | + | - | ND | + |
| quinic acid | + | ND | + | + | + | + | ND | + |
| D-saccharic acid | + | ND | - | + | - | - | ND | + |
| vancomycin | + | ND | + | + | + | + | ND | + |
| tetrazolium violet | + | ND | + | + | + | + | ND | + |
| tetrazolium blue | + | ND | + | + | + | + | ND | + |
| p-hydroxy-phenylacetic acid | - | ND | - | + | + | + | ND | - |
| methyl pyruvate | + | ND | + | + | + | + | ND | + |
| D-lactic acid methyl ester | + | ND | - | - | + | + | ND | + |
| L-lactic acid | + | ND | + | + | + | + | ND | + |
| citric acid | + | ND | + | + | + | + | ND | + |
| α-keto-glutaric acid | - | ND | - | - | - | - | ND | - |
| D-malic acid | - | ND | - | - | - | - | ND | - |
| L-malic acid | + | ND | + | + | + | + | ND | + |
| bromo-succinic acid | + | ND | + | + | + | + | ND | + |
| nalidixic acid | + | ND | + | + | + | + | ND | + |
| lithium chloride | + | ND | + | + | + | + | ND | + |
| potassium tellurite | - | ND | - | - | - | - | ND | - |
| tween 40 | + | ND | - | - | + | + | ND | + |
| g-amino-butyric acid | + | ND | - | - | - | - | ND | - |
| α-hydroxy-butyric acid | + | ND | - | - | - | - | ND | - |
| β-hydroxy-D,L-butyric acid | + | ND | - | - | - | - | ND | - |
| α-keto-butyric acid | - | ND | - | - | - | - | ND | - |
| acetoacetic acid | + | ND | - | - | + | + | ND | - |
| propionic acid | - | ND | - | - | - | - | ND | - |
| acetic acid | + | ND | + | + | + | + | ND | + |
| formic acid | - | ND | - | - | - | - | ND | - |
| aztreonam | + | ND | + | + | + | + | ND | + |
| sodium butyrate | + | ND | + | + | + | + | ND | + |
| sodium bromate | - | ND | - | - | - | - | ND | - |

+ = positive, - = negative, ND = not determined.
